# Supplementary material for: Localization and Single Molecule Dynamics of Bacillus subtilis Penicillin-Binding Proteins Depend on Substrate Availability and Are Affected by Stress Conditions
Source: Cells. 2025 Mar 13;14(6):429. doi: 10.3390/cells14060429 (PMC11940910; doi:10.3390/cells14060429)
Supplement: Supplementary file 1 [file cells-14-00429-s001.zip › cells-3434164-supplementary.pdf]

Localization and single molecule dynamics of *Bacillus subtilis* Penicillin binding proteins depend on substrate availability and are affected by stress conditions

Lisa Stuckenschneider and Peter L. Graumann

SYNMIKRO, LOEWE-Zentrum für Synthetische Mikrobiologie, Hans-Meerwein-Straße, 35043 Marburg, and Fachbereich Chemie, Hans-Meerwein-Straße 4, 35032 Marburg, Germany

Supplementary data

Movie S1: exponentially growing *B. subtilis* cells expressing mVenus-Pbp2a from the original gene locus. 20 ms stream acquisition, shown are 50 frames/s.

Movie S2: *B. subtilis* cells expressing mVenus-Pbp2a from the original gene locus, 30 minutes after addition of 4 µg/ml of Vancomycin to a growing culture. 20 ms stream acquisition, shown are 50 frames/s.

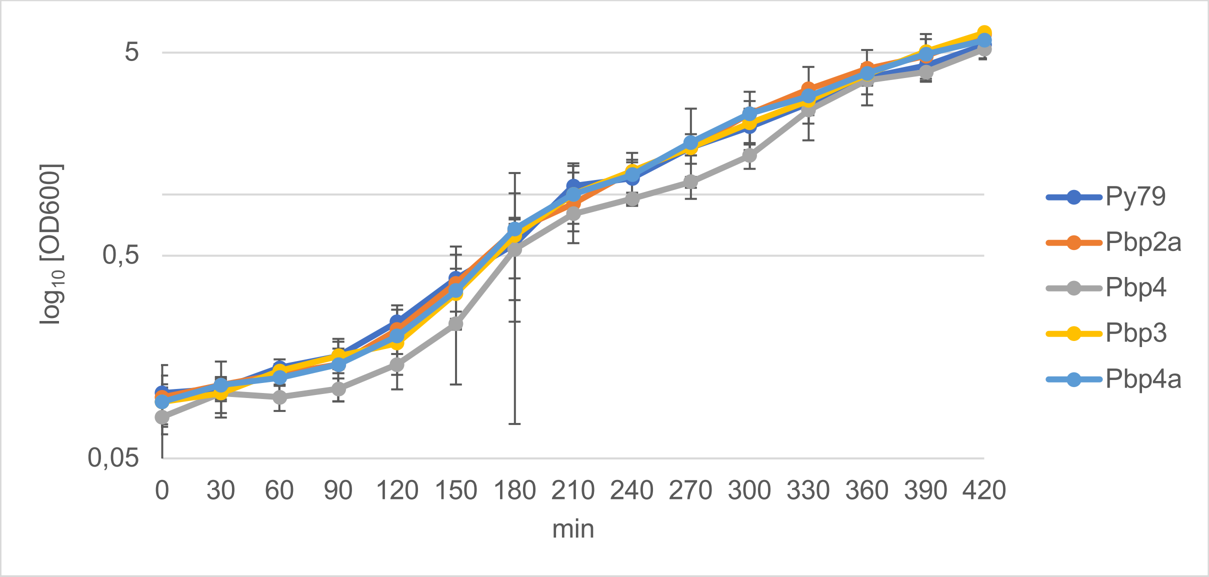

Figure S1 Growth curves of mVenus -PBP fusion strains compared to *Bacillus subtilis* PY79 wild type cells. The growth experiment was performed in 50 ml LB medium with 0.01% xylose for the mVenus-PBP fusion strains as a duplicate starting with an initial optical density (OD_{600}) of 0.1 (t_0). Measuring the OD_{600} every 30 minutes for 7 hours.

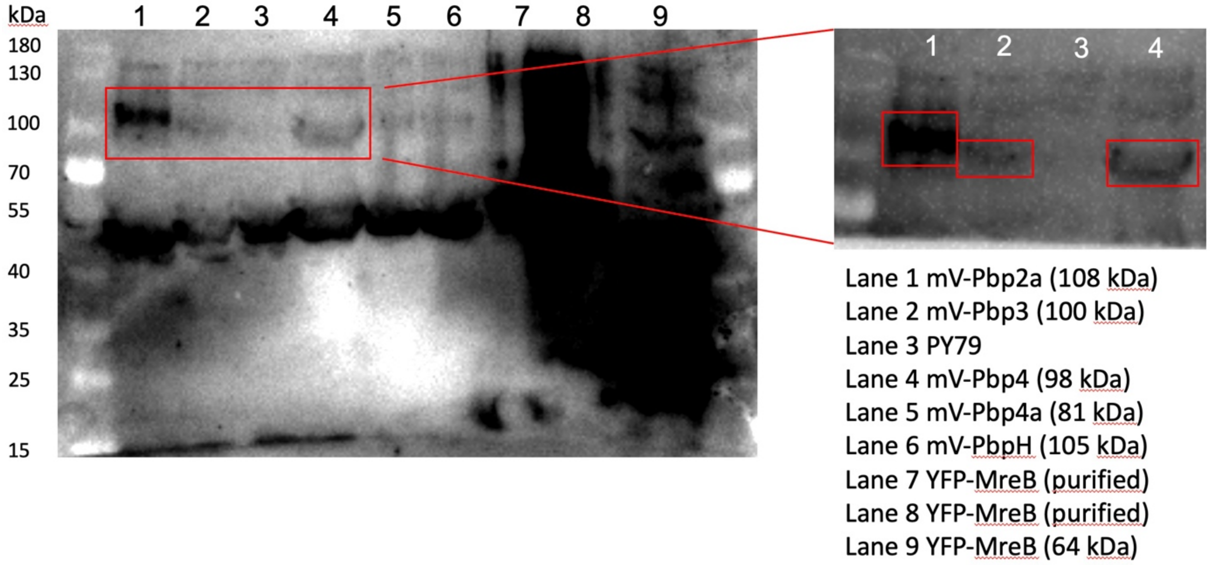

Figure S2 Western blot analysis of the mVenus-PBP fusion strains with an anti-GFP antibody; the nature of the cell extract (or purified protein, lanes 7 and 8) is indicated in the legend on the right. Purified YFP-MreB was used as a positive control for antibody binding, and *B. subtilis* PY79 was used to indicate unspecific binding of the antibody. Expected bands are marked with red boxes in the inset on the right. Note that strains expressing mVenus(mV)-PBP fusions were grown using 0.5% xylose in the medium, resulting in full induction of the promoter driving transcription of genes encoding mV-PBPs. mV-PBPs were undetectable in Western blots using 0.01% xylose induction that was used for imaging experiments. Note that this low level of induction was sufficient to sustain viability in mutant strains (see Fig. S3).

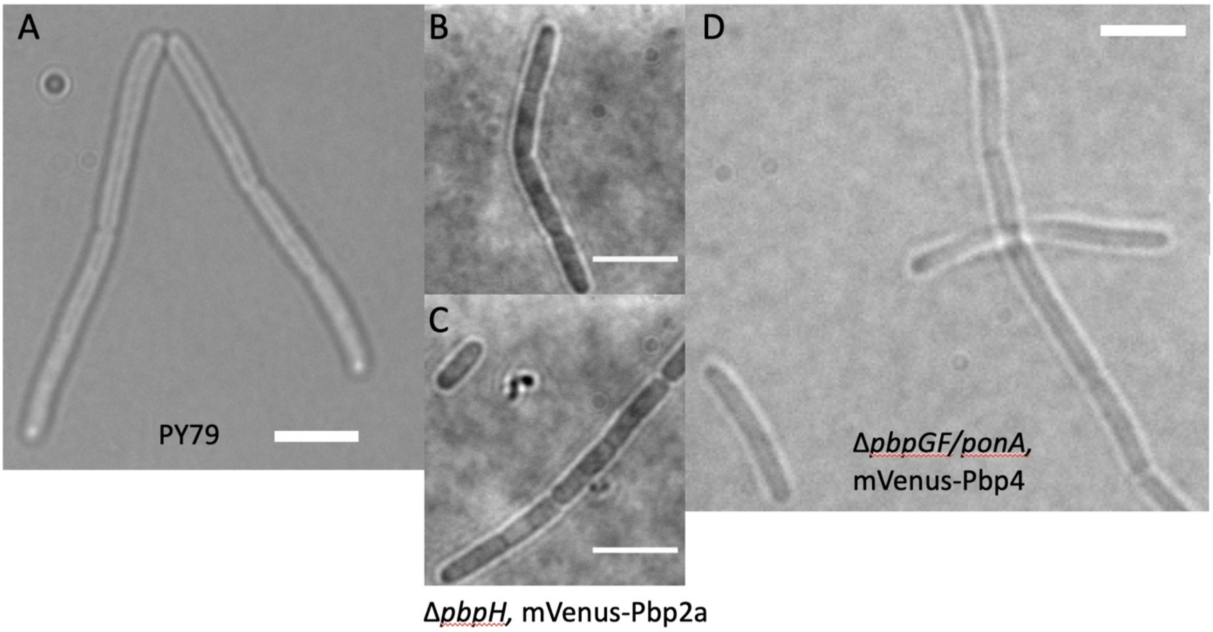

Figure S3 Brightfield pictures of mVenus-fusions with a deletional background to provide evidence for functionality. A) wild type cells, B-C) Δ*pbpH,* mVenus-Pbp2a cells, D) Δ*pbpGF/ponA,* mVenus-Pbp4 cells. Cells were cultured in liquid LB medium, containing 0.01% xylose in case of mutant strains carrying PBP fusion constructs (panels B-D) until an OD_{600} 0.6. Scale bars 3 µm.

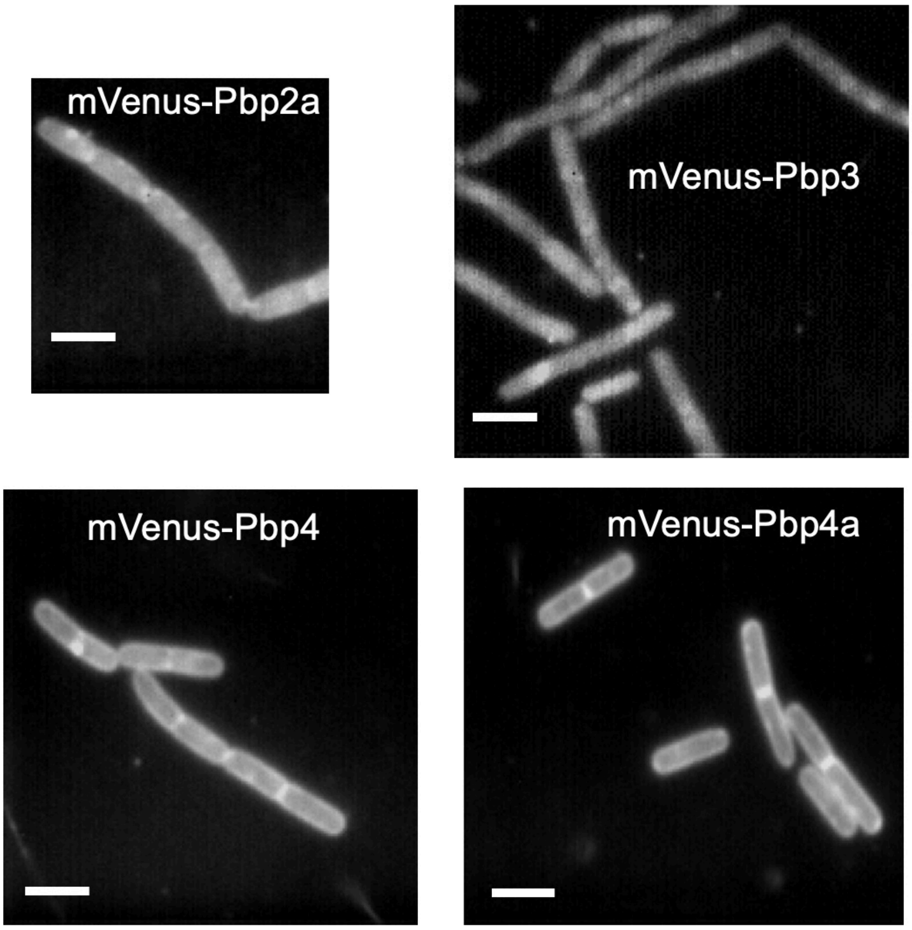

Figure S4 Fluorescent micrographs corresponding to Fig. 1B with equal scaling, scale bars 2 µm.

Table S1: List of strains

Strain	Genotyp	Resistance	Source
***E. coli* DH5α**	Wild type	-	
***Bacillus subtilis* PY79**	Wild type	-	
***E. coli* pHJDS-mVenus**	Plasmid pHJDS-P_{xyl}-mVenus	amp	This study
**PY79 mV-Pbp2A**	P_{xyl}- *mV-pbpA* (original locus)	Cm	This study
**PY79 mV-Pbp3**	P_{xyl}- *mV-pbpC* (original locus)	Cm	This study
**PY79 mV-Pbp4**	P_{xyl}- *mV-pbpD* (original locus)	Cm	This study
**PY79 mV-Pbp4a**	P_{xyl}- *mV-dacC* (original locus)	Cm	This study
**PY79 ΔpbpH mV-Pbp2A**	P_{xyl}- *mV-pbpA* (original locus), pbpH::kan^R	Cm, kan	This study
**168 ΔpbpGFponA mV-Pbp4**	P_{xyl}- *mV-pbpD* (original locus), pbpG and pbpF markerless deletion, ponA::kan^R	Cm, kan	This study

Table S2: Table of detailed information generated by the SMTracker data analysis. Data complementary to Fig. 2.

**Comparison of Pbps**	Pbp2a	Pbp3	Pbp4	Pbp4a
# movies	33	29	24	32
# cells	208	230	165	271
av. cell length [µm]	2.8700	3.1600	3.1200	2.6700
# tracks	2030	943	2969	1844
#tracks/cell	9.7596	4.1000	17.9939	6.8044
Dwell time radius [nm]	120	120	120	120
static tracks [%]	1.9000	4.1000	2	3.3000
mobile tracks [%]	98.1000	95.9000	98	96.7000
free [%]	94.9000	91.5000	94.2000	93.4000
mixed behaviour [%]	3.2000	4.3000	3.8000	3.3000
**Diffusion constants from SQD and JD**				
pop_1 [%]	47.4 ± 0.002	52.3 ± 0.002	42.6 ± 0.001	48.8 ± 0.002
pop_2 [%]	52.6 ± 0.002	47.7 ± 0.002	57.4 ± 0.001	51.2 ± 0.002
D_1 [$\mu m^2 s^{-1}$]	0.081 ± 0	0.067 ± 0	0.093 ± 0	0.067 ± 0
D_2 [$\mu m^2 s^{-1}$]	0.57 ± 0.002	0.56 ± 0.003	0.62 ± 0.002	0.55 ± 0.003
**Dwell times**				
Ꞇ (1-comp.) ± sd [ms]	0.27 ± 0.007 s	0.26 ± 0.004 s	0.31 ± 0.006 s	0.3 ± 0.01 s
stars / p-value	**Pbp2a**	**Pbp3**	**Pbp4**	**Pbp4a**
**Pbp2a**	-	(lv)* / 0.057814	(lv)* / 0.074121	(lv) *** / 0.00047204
**Pbp3**	-	-	(lv) *** / 0.00038642	(lv) *** / 1.3537e-05
**Pbp4**	-	-	-	(lv) *** / 0.0020699
**Pbp4a**	-	-	-	-

Table S3: Table of detailed information generated by the SMTracker data analysis. Data complementary to Fig. 3.

**Condition of strain mV-Pbp2a**	Non stressed	500 mM NaCl	1 M sorbitol	4 µg/ml vancomycin	4 µg/ml penicillin G
# movies	33	30	30	27	28
# cells	232	188	192	222	217
av. cell length [µm]	2.8200	3.1400	2.9800	3.2600	3.0900
# tracks	2030	3619	1673	8116	2976
#tracks/cell	10.1400	21.1548	9.1803	38.5856	15.5370
dwell time radius [nm]	120	120	120	120	120
static tracks [%]	1.9000	2.2000	2.7000	2.5000	3.5000
mobile tracks [%]	98.1000	97.8000	97.3000	97.5000	96.5000
free [%]	94.9000	93.8000	93.7000	94.4000	91.4000
mixed behaviour [%]	3.2000	4	3.6000	3.1000	5.2000
**Diffusion**	**constants**	**from**	**GMM**		
Static D ± sd [$\mu m^2 s^{-1}$]	0.077 ± 0.00027	0.077 ± 0.00027	0.077 ± 0.00027	0.077 ± 0.00027	0.077 ± 0.00027
Mobile D ± sd [$\mu m^2 s^{-1}$]	0.71 ± 0.0021	0.71 ± 0.0021	0.71 ± 0.0021	0.71 ± 0.0021	0.71 ± 0.0021
Static fraction ± sd [%]	50.3 ± 0.14	45.8 ± 0.13	52.8 ± 0.14	35.8 ± 0.12	50.8 ± 0.13
Mobile fraction ± sd [%]	49.7 ± 0.14	54.2 ± 0.13	47.2 ± 0.14	64.2 ± 0.12	49.2 ± 0.13
**Dwell times**					
τ (1-comp.) ± sd [ms]	0.26 ± 0.0066 s	0.27 ± 0.0035 s	0.28 ± 0.0088 s	0.29 ± 0.0054 s	0.32 ± 0.008 s
stars / p-value	**Non stressed**	**500 mM NaCl**	**1 M sorbitol**	**4 µg/ml vancomycin**	**4 µg/ml penicillin G**
Non-stressed	-	(tt) ns / 0.96507	(lv) *** / 0.008692	(tt) ns / 0.2015	(lv) *** / 0.00058067
500 mM NaCl	-	-	(lv) *** / 1.4291e-05	(lv) *** / 0.0011055	(lv) *** / 5.3127e-09
1 M sorbitol	-	-	-	(lv) ** / 0.022889	(tt) ns / 0.23315
4 µg/ml vancomycin	-	-	-	-	(lv) *** / 5.6086e-05

4 µg/ml penicillin G	-	-	-	-	-

Table S4: Table of detailed information generated by the SMTracker data analysis. Data complementary to Fig. 4.

**Condition of strain mV-Pbp3**	Non stressed	500 mM NaCl	1 M sorbitol	4 µg/ml vancomycin	4 µg/ml penicillin G
# movies	29	29	28	24	26
# cells	277	216	179	202	180
av. cell length [µm]	3.1400	3.1700	3.5400	3.4500	3.1400
# tracks	943	884	1610	6860	1511
#tracks/cell	4.2130	4.8309	9.9787	39.3305	8.7685
dwell time radius [nm]	120	120	120	120	120
static tracks [%]	4.1000	6.4000	1.7000	3.3000	3.3000
mobile tracks [%]	95.9000	93.6000	98.3000	96.7000	96.7000
free [%]	91.5000	88.6000	94.3000	93.2000	93.6000
mixed behaviour [%]	4.3000	5	3.9000	3.5000	3
**Diffusion constants from GMM**					
Static D ± sd [$\mu m^2 s^{-1}$]	0.063 ± 0.0002	0.063 ± 0.0002	0.063 ± 0.0002	0.063 ± 0.0002	0.063 ± 0.0002
Mobile D ± sd [$\mu m^2 s^{-1}$]	0.72 ± 0.002	0.72 ± 0.002	0.72 ± 0.002	0.72 ± 0.002	0.72 ± 0.002
Static fraction ± sd [%]	55.4 ± 0.12	59.2 ± 0.12	46.6 ± 0.11	41.7 ± 0.1	43.3 ± 0.11
Mobile fraction ± sd [%]	44.6 ± 0.12	40.8 ± 0.12	53.4 ± 0.11	58.3 ± 0.1	56.7 ± 0.11
**Dwell times**					
Ꞇ (1-comp.) ± sd [ms]	0.26 ± 0.0038 s	0.27 ± 0.0042 s	0.25 ± 0.0047 s	0.28 ± 0.0028 s	0.34 ± 0.0071 s
stars / p-value	**Non stressed**	**500 mM NaCl**	**1 M sorbitol**	**4 µg/ml vancomycin**	**4 µg/ml penicillin G**
**Non-stressed**	-	(tt) ns / 0.55131	(tt) ns / 0.69622	(lv)* / 0.055643	(lv) *** / 0.00013165
**500 mM NaCl**	-	-	(tt) ns / 0.97333	(tt) ns / 0.41856	(lv) *** / 0.00084379
**1 M sorbitol**	-	-	-	(tt) ns / 0.43051	(lv) ** / 0.034142

**4 µg/ml vancomycin**	-	-	-	-	(lv) *** / 0.0048857
**4 µg/ml penicillin G**	-	-	-	-	-

Table S5: Table of detailed information generated by the SMTracker data analysis. Data complementary to Fig. 5.

**Condition of strain mV-Pbp4**	Non stressed	500 mM NaCl	1 M sorbitol	4 µg/ml vancomycin	4 µg/ml penicillin G
# movies	24	30	24	25	26
# cells	256	292	265	275	221
av. cell length [µm]	3.06	3.1	3.11	3.47	3.31
# tracks	2969	7377	1463	2551	2902
#tracks/cell	17.8	41.4	6.5	58.3	14.3
dwell time radius[nm]	120	120	120	120	120
static tracks [%]	2	1.9	4	3	4.4
mobile tracks [%]	98	98.1	96	97	95.6
free [%]	94.2	93.4	90.8	93.9	91.7
mixed behaviour [%]	3.8	4.6	5.3	3.2	3.9
**Diffusion constants from GMM**					
Static D ± sd [$\mu m^2 s^{-1}$]	0.063 ± 0.0003	0.063 ± 0.0003	0.063 ± 0.0003	0.063 ± 0.0003	0.063 ± 0.0003
Mobile D ± sd [$\mu m^2 s^{-1}$]	0.73 ± 0.002	0.73 ± 0.002	0.73 ± 0.002	0.73 ± 0.002	0.73 ± 0.002
Static fraction ± sd [%]	39.7 ± 0.14	41.4 ± 0.13	59.6 ± 0.17	40.4 ± 0.13	46.7 ± 0.15
Mobile fraction ± sd [%]	60.3 ± 0.14	58.6 ± 0.13	40.4 ± 0.17	59.6 ± 0.13	53.3 ± 0.15
**Dwell times**					
τ (1-comp.) ± sd [s]	0.31 ± 0.0063 s	0.29 ± 0.004 s	0.33 ± 0.0064 s	0.3 ± 0.0042 s	0.28 ± 0.0057 s
stars / p-value	**Non stressed**	**500 mM NaCl**	**1 M sorbitol**	**4 µg/ml vancomycin**	**4 µg/ml penicillin G**
**Non-stressed**	-	(tt) ns / 0.40334	(lv)* / 0.052432	(tt) ns / 0.6974	(tt) ns / 0.65515

**500 mM NaCl**	-	-	(lv) *** / 0.0072279	(lv) ** / 0.023322	(tt) ns / 0.80676
**1 M sorbitol**	-	-	-	(tt) ns / 0.37461	(tt) ns / 0.15913
**4 µg/ml vancomycin**	-	-	-	-	(tt) ns / 0.35744
**4 µg/ml penicillin G**	-	-	-	-	-

Table S6: Table of detailed information generated by the SMTracker data analysis. Data complementary to Fig. 6.

**Condition of strain mV-Pbp4a**	Non stressed	500 mM NaCl	1 M sorbitol	4 µg/ml vancomycin	4 µg/ml penicillin G
# movies	32	38	37	36	25
# cells	322	307	350	218	179
av. cell length [µm]	2.6500	2.8500	2.7600	3.4100	3.0700
# tracks	1844	6246	4270	15923	1856
#tracks/cell	7.1800	24.1588	20.2282	69.9525	10.8275
dwell time radius [nm]	120	120	120	120	120
static tracks [%]	3.3000	3.6000	3.9000	2.8000	2.1000
mobile tracks [%]	96.7000	96.4000	96.1000	97.2000	97.9000
free [%]	93.4000	88.9000	89.4000	93.5000	94.2000
mixed behaviour [%]	3.3000	7.4000	6.7000	3.7000	3.7000
**Diffusion constants from GMM**					
Static D ± sd [μm^2 s^{-1}]	0.091 ± 0.00033	0.091 ± 0.00033	0.091 ± 0.00033	0.091 ± 0.00033	0.091 ± 0.00033
Mobile D ± sd [μm^2 s^{-1}]	0.89 ± 0.0048	0.89 ± 0.0048	0.89 ± 0.0048	0.89 ± 0.0048	0.89 ± 0.0048
Static fraction ± sd [%]	64.8 ± 0.19	64.8 ± 0.17	60.9 ± 0.17	51.4 ± 0.17	47.9 ± 0.2
Mobile fraction ± sd [%]	35.2 ± 0.19	35.2 ± 0.17	39.1 ± 0.17	48.6 ± 0.17	52.1 ± 0.2
**Significantly different dwell times**					
Ƈ (1-comp.) ± sd [s]	0.3 ± 0.011 s	0.28 ± 0.0042 s	0.28 ± 0.0035 s	0.27 ± 0.0033 s	0.27 ± 0.0029 s

stars / p-value	**Non stressed**	**500 mM NaCl**	**1 M sorbitol**	**4 µg/ml vancomycin**	**4 µg/ml penicillin G**
**Non-stressed**	-	(lv) *** / 2.7843e-05	(lv) *** / 1.2465e-09	(lv) *** / 0.00023352	(lv) ** / 0.015592
**500 mM NaCl**	-	-	(lv) ** / 0.022042	(tt) ns / 0.82601	(tt) ns / 0.8092
**1 M sorbitol**	-	-	-	(lv) ** / 0.037105	(tt) ns / 0.7364
**4 µg/ml vancomycin**	-	-	-	-	(tt) ns / 0.90286
**4 µg/ml penicillin G**	-	-	-	-	-
